# A multicomponent family intervention, combined with salt reduction for children with obesity: a factorial randomized study protocol

**DOI:** 10.1186/s12889-023-16356-6

**Published:** 2023-07-29

**Authors:** Cinthia Guimarães Assemany, Diana Barbosa Cunha, Joana Maia Brandão, Vitor Barreto Paravidino, Magno Conceição Garcia, Ana Lúcia Viégas Rêgo, Rosangela Alves Pereira, Rosely Sichieri

**Affiliations:** 1 Department of Epidemiology, Institute of Social Medicine, State University of Rio de Janeiro, CEP 20550-013 Rio de Janeiro, Brazil; 2 Department of Social and Applied Nutrition, Federal University of Rio de Janeiro, Rio de Janeiro, Brazil

**Keywords:** Pediatric obesity, Diet, Healthy, Medicinal herbs, Diet, Sodium-restricted

## Abstract

**Background:**

Clinical trials to treat childhood obesity show modest results, weight regain and high dropout rates. Children with obesity often live in families with habits that contribute to unhealthy weight gain. This study will test whether a family intervention with a Brazilian-adapted Planetary Healthy Diet (PHD) and reduced portion sizes, along with increased physical activity and reduced sedentary behavior, can reduce excessive weight gain. The protocol promotes the intake of in natura products and water and reduces ultra-processed foods, sugar, and sodium. It encourages family lifestyle changes and physical activities, with randomized allocation to experimental and control groups. The responsible family member will be evaluated during follow-up. The control group will receive a print of the Brazilian dietary guideline.

**Methods:**

A factorial crossover design will also allocate families to receive reduced sodium salt plus anti-inflammatory herbs and a placebo salt. Both the control and intervention groups will be randomly assigned to the sequence of both salts. The approach aims to reduce body weight expectations and evaluate salt's impact on blood pressure. It includes a 1-month intervention, 1-month washout, and 1-month intervention with monthly clinic visits and teleservice by health professionals. The primary outcomes will be the variation in the Body Mass Index (BMI) of the children. BMI and the variation in the blood pressure of the pair (child/mother or father) as well as waist circumference (WC) and waist-to-height ratio (WHtR) will also be measured.

**Discussion:**

The project will test the effectiveness of the use of the recommendations of the PHD, physical activity and a salt-reduced sodium. The results of the present study will allow the refinement of interventions aimed at the treatment of childhood obesity and may help develop guidelines for the treatment of obesity in Brazilian children.

**Trial registration:**

The study is registered in the Brazilian Registry of Clinical Trials (RBR-10 mm62vs). Registered 10 February 2023.

## Introduction

Interventions to treat obesity with a primary focus on weight change show modest results [1]. The position paper “Clinical Practice Guideline for the Evaluation and Treatment of Children and Adolescents with Obesity” by the American Academy of Pediatrics, published in February 2023 [2], reinforces the role of a coordinated, family- and child-centered approach to treat childhood obesity, with a focus on lifestyle behavior change. A systematic review found that parental involvement through goal setting, counseling, role modeling, and restructuring the environment produced changes in nutritional behavior and physical activity in children aged 7–13 years [3].

A Brazilian study conducted with children aged 6 to 11 years found that a 100 g increase in the contribution of ultra-processed foods to daily food intake was associated with a gain of 0.14 kg/m^2^ in fat mass index, mediated by its caloric content [4]. Additionally, a cross-sectional study with children eight to 12 years from public schools in southeastern Brazil showed that the intake of ultra-processed foods was positively associated with overweight/obesity [5]. Changes in the consumption of ultra-processed products are a promising strategy for reducing excessive weight gain in children, but further control of energy intake increases weight reduction, as shown in a controlled trial [6]. Reduction of portion sizes is a pathway to reduce food and energy intake, and this strategy includes the use of tall, thin mugs and cups with small volumes, as well as smaller diameter dishes, bowls, and utensils, such as dishes with hoops that limit the internal space [7].

The diet should also promote environmental sustainability, in addition to nutritional value. The Planetary Healthy Diet (PHD) achieves this by minimizing ultra-processed foods, increasing fresh foods and whole grains, limiting animal-based foods, and using sustainably produced vegetable fats [8]. Developed by the EAT-Lancet Commission, the PHD can be adapted to national consumption patterns, as shown by Marchioni et al., who proposed the Brazilian Planetary Health Diet Index (PHDI). PHDI was associated with higher diet quality and lower greenhouse gas emissions [9]. Cross-sectional data from the Longitudinal Study of Adult Health (ELSA-Brazil) showed that individuals with greater adherence to the PHD-adapted diet had lower body mass index (BMI) and waist circumference (WC), were less likely to be overweight or obese, regardless of sociodemographic characteristics [10] and had lower levels of blood pressure, total cholesterol, LDL-c, and non-HDL-c [11].

A specific aspect of the Brazilian diet is the high intake of table salt [12], one of the main dietary risk factors for chronic diseases [13]. Multiple physiological mechanisms related to hormonal factors, inflammation, the immune response and the intestinal microbiome are involved in the various effects of excessive sodium consumption in the body [14]. The substitution of regular salt with light salt, where sodium chloride in traditional salt is partially replaced by potassium chloride or magnesium sulfate, is considered a strategy by several countries for the reduction of blood pressure and stroke [15]. A randomized controlled trial investigated the effects of replacing common salt with light salt for 3 years in a rural population of northern China. Light salt significantly reduced the increase in systolic and diastolic blood pressure (BP) compared to regular salt [16]. A limitation for the use of this product is the change in the taste of food, decreasing adherence. The addition of herbs, making the product tastier, increased acceptability [17–19].

The herbs added to the salt in this project are the ones with the greatest anti-inflammatory effect among the most commonly used herbs in Brazil. Medicinal plants or their constituents are considered beneficial due to the presence of phytoconstituents that can prevent undesirable inflammatory processes, in addition to the ease of availability, low cost, and absence of side effects [20, 21].

Childhood is a crucial period for establishing healthy habits and increasing physical activity [22], but physical inactivity and sedentary behavior are on the rise [23]. Studies show that physical activity can improve BMI and prevent childhood obesity, particularly when parents also participate in interventions [22]. This is in line with the evolution of social cognitive theory, which recognizes children as agents of their behavior that are influenced by personal and family factors [24].

This study aims to determine the impact of a comprehensive family-based approach on reducing children's weight gain. The intervention includes guidance on sustainable eating habits, reducing ultra-processed foods and sodium, increasing consumption of brown rice and seeds, but reducing portion sizes. It will also promote physical activity and reduce sedentary behavior. The reduction in sodium intake may also contribute to lowering blood pressure and managing family expectations regarding weight loss.

## Methods and study design

### Objectives

#### Primary objective

Determine whether a factorial design of dietary intervention based on the PHD combined with physical exercises performed with family members reduces excessive weight gain in children with obesity.

#### Secondary objectives

1. Determine whether the use of low-sodium salt added to anti-inflammatory herbs reduces the blood pressure of child-guardian pairs.
2. Determine if the family intervention promoted weight loss of the child-guardians.
3. Investigate the effect of the intervention on total physical activity and sedentary time in children and guardians.

### Design of the study

**Fig. 1:**
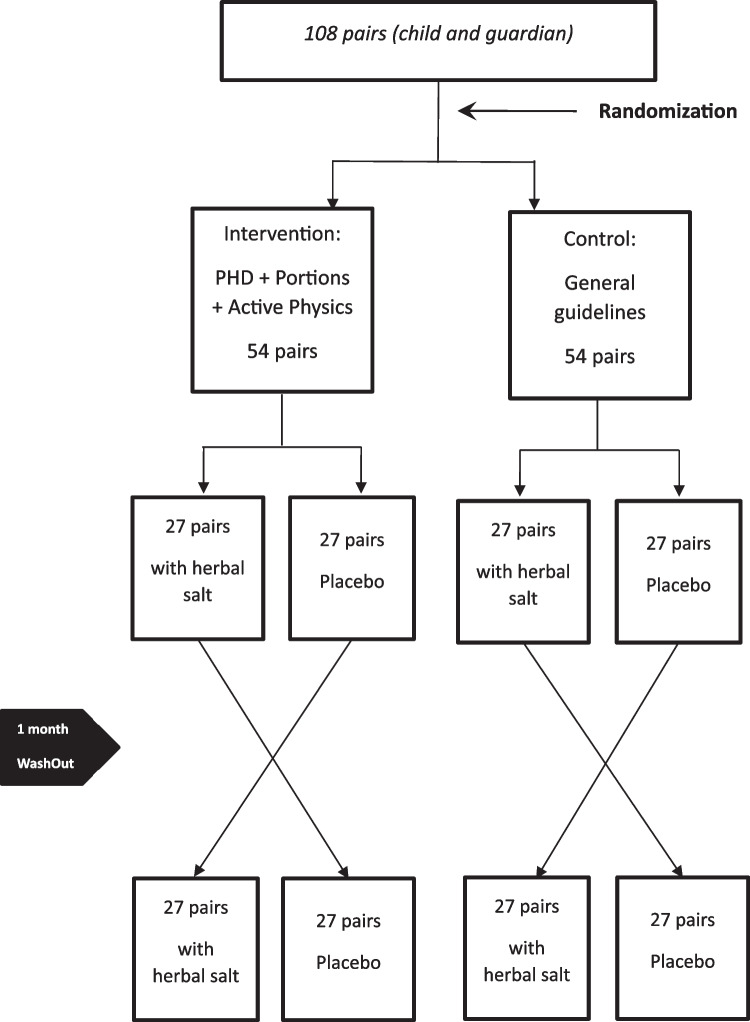
Flowchart of the study

Factorial randomized clinical trial for the treatment of obesity with children between 7 and 12 years of age. Children and one of their guardians will be randomized to intervention with guidance regarding diet based on the PHD, on portion size reduction and a physical exercise program or control with general healthy eating guidance for a period of three months. The guidelines will be directed to all family members. Additionally, pairs in each arm will cross-receive a low-sodium salt added anti-inflammatory spices compared to a placebo salt (normal sodium content). The groups will be randomly subdivided into 4 groups of 27 individuals: 1 subgroup of the intervention group and 1 subgroup of the control group will receive hyposodium herbal salt, and the other 2 groups will receive placebo for 1 month. After 1 month of washout, the groups will receive the salt cross-label (Fig. 1). The protocol is reported according to the Standard Protocol Items: Recommendations for Interventional Trials (SPIRIT) [25].

### Recruitment and selection

#### Inclusion and exclusion criteria

Recruitment for the study will be conducted through various channels, including schools in Rio de Janeiro, a public university's banner and radio, and social media platforms. Interested participants can register by emailing their information based on the eligibility criteria. Once selected, participants will be scheduled for an initial face-to-face consultation where they will receive all relevant information about the project and provide their informed consent.

Families with at least one child aged 7 to 12 years with overweight or obesity (Z score 1.5) and who make at least 1 meal at home per day will be included in the study.

The exclusion criterion will be based on the known preexistence of genetic disorders or endocrine disorders associated with obesity, as well as children with some disability that prevents anthropometric measurements. Patients using weight loss medications and in continuous corticosteroid use and families that have at least one member who has an allergy or some other restriction to some ingredient in hyposodium herbal salt will be excluded. All this information will be obtained through a questionnaire completed by the guardian and the child.

### Sample size and randomization procedure

The sample size of 48 pairs (child and one guardian) per group was calculated from a standard deviation for BMI equal to 3.0 and an expected difference of 1.72 units of BMI in the child [26] between the groups, considering a statistical power of 80% and significance level of 5%. This sample size of 96 pairs, 192 individuals plus an expected dropout of 10% of the participants make a final sample of 108 pairs (54 pairs in each group). After the recruitment phase, participants considered eligible will be randomly allocated to the control group or intervention group. The randomization will be performed in blocks of 8 individuals using a sequence of random numbers generated by a computer.

The necessary sample size for changes in blood pressure was based on data for adults (the guardians) of the *DASH (*Dietary Approaches to Stop Hypertension (Sacks et al.) and a Brazilian dietary intervention trial for adults [27]. The Brazilian trial focused on salt intake reduction, and after 6 months, systolic blood pressure was reduced by 14·4 mmHg and diastolic blood pressure by 9·7 mmHg in the experimental group compared with 6·7 and 4·6 mmHg, respectively, in the control group. A sample size of thirty-one subjects per arm (total 62) assumed a reduction in systolic blood pressure of 7·1 mmHg and a standard deviation of 10 mmHg [28] with a significance level of 5% and 80% power.

Therefore, the sample size for the change in BMI also allowed us to evaluate changes in blood pressure, at least for adults.

We used sequentially numbered, opaque paper bags with the salt and utensils given to the children´s family to preserve allocation concealment.

### Intervention

**Fig. 2:**
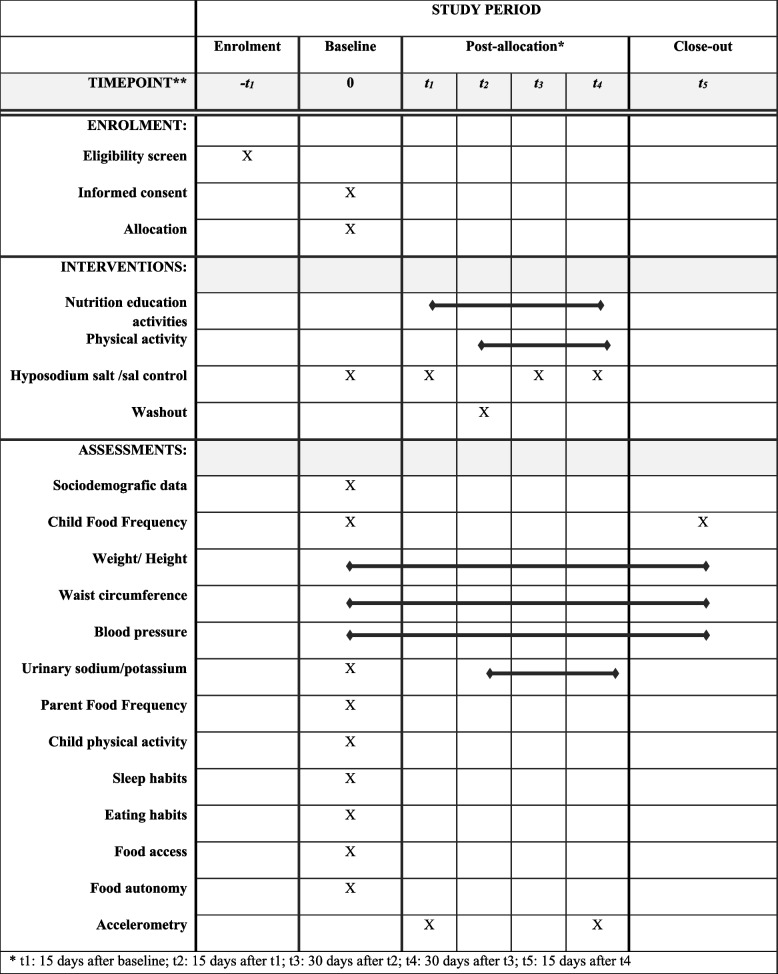
Overview of the study period (SPIRIT Fig. 2013)

The intervention will take place through face-to-face consultations in a reserved room at the State University of Rio de Janeiro by a team of nutritionists and physical education teachers. There will be 6 consultations. The first and the last with a 15-day break. The others were carried out monthly. The timeline of the interventions and the instruments used in the initial measurement and follow-up are described below in the SPIRIT timetable of the study presented in Fig. 2.

#### Nutritional guidelines on the healthy planetary diet

**Fig. 3:**
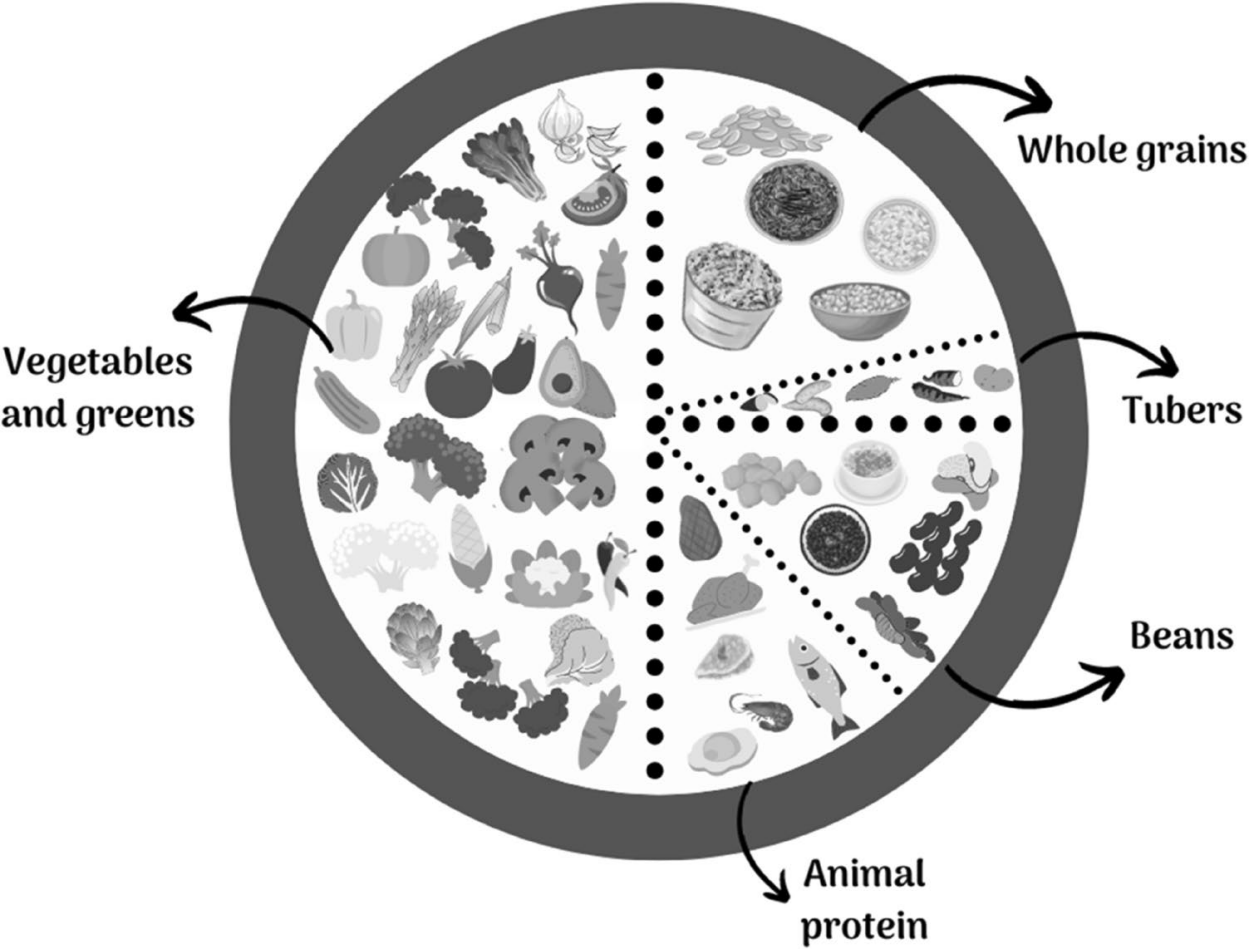
Adapted plan of the Planetary Healthy Diet for the Brazilian diet

The nutritional guidelines will be based on the items that make up the Planetary Diet Index. They are nuts and peanuts; vegetables, fruits, and vegetables; whole grains; eggs; fish; tubers; milk and dairy products; vegetable oils; red meat; chicken; animal fat and added sugar. An adaptation of the proposal that will be used can be seen in Fig. 3.

As an incentive, the intervention group will receive throughout the intervention period a cookbook, a calendar of food goals, a booklet of guidance in relation to food, guidance regarding the purchase of healthy foods produced locally, the importance of buying food from the crop, and reducing loss and waste and reading labels.

Through a Food Frequency Questionnaire (FFQ), we will be able to better understand the daily life of the family and thus establish goals that the family must achieve, which will help adherence to PHD. Each month, the child and his family will be encouraged to choose one of the following goals: 1) reduce the consumption of ultra-processed foods, 2) increase the consumption of nuts, 3) try recipes with full use of foods, 4) reduce the consumption of red meat, 5) increase the consumption of whole grains, 6) increase the consumption of fruits, and 7) increase the consumption of vegetables and greens. A calendar will be provided every month, and stickers will be provided for the family to fill the days they can achieve the goals proposed by the professionals. It is a playful way for the child and their guardian to be encouraged to follow the proposed guidelines.

In addition to the goals, each meeting will be held as an educational activity with the child and their guardian. The first has already been tested by the research group in a previous study and is based on the degree of food processing and encouragement to purchase food at street markets [29]. The second activity aims to increase and diversify the consumption of fresh food purchased at street markets, encouraging a more active participation of children in their diet. The third activity is related to the consumption of whole grains. In addition, recipes and ludic activities to be carried out at home will be delivered to the participants.

The control group will receive general healthy eating guidelines based on those promoted in primary care for children with obesity and will also receive hyposodium herbal salt in the crossover design. Materials developed for the experimental group will be given to the control group at the end of the study.

#### Hyposodium salt

Herbal salt is composed of 25% potassium chloride (KCl) and 10% a mixture of rosemary, turmeric, basil, mint and oregano and will be delivered along with information on how to use it on the day of the first appointment. The placebo salt is a combination of regular salt and pink Himalaya salt.

#### Reduction of portion sizes

Children in the intervention group will be provided with utensils that encourage them to reduce the size of their portions: a soup plate with a colored border, a long and narrow glass and a bowl of reduced size [7]. Children will be instructed to eat all meals, when possible, on the utensils received.

Both the offer of salt and utensils aim to stimulate the domestic production of food, and, periodically, simple recipes will be offered to families.

#### Physical activity and sedentary behavior

The participants in the intervention group will receive a dance choreography video plus two or three gym class links available on YouTube each week to increase moderate-to-vigorous physical activity. On average, the duration of the video classes is 45 min. At the beginning of the intervention, a kit of sports materials containing ropes and balls and a booklet with information about the guidelines on physical activity and sedentary behavior for children and adolescents will be provided.

To improve adherence to the intervention, a set of motivational messages will be sent each week via WhatsApp, encouraging children to perform the activities. Additionally, children and their guardians will receive a list of goals to reduce sedentary time and increase physical activity throughout the research period, and they will choose two of them to achieve each week.

### Data analysis

The rate of change in the primary and secondary outcomes over time will be investigated by intention-to-treat analysis using mixed-effects models, which allow us to consider incomplete follow-up data and the correlation of repeated measures. The verification of the adequacy of the models will be done graphically through the diagnosis of the residues [30]. SAS software, version 9.4 (SAS Institute, Cary, NC, USA) will be used. In secondary analyses, with measurements of more than one family member, the correlation of the family cluster in the change estimates will be considered.

### Outcomes

#### Primary outcome

The primary outcome of the study will be the variation in the children's body mass index (BMI). Body weight will be measured on a portable electronic scale (Tanita BC-558). Height will be measured in duplicate with the use of a portable anthropometer of the brand Altura Exata. Both measurements will be measured with the assessed, barefoot, wearing light clothing, without props in the hair, with the arms extended to the side of the body, positioned by the horizontal plane of Frankfurt. The classification of nutritional status will be performed based on BMI/age values, in z scores, using the new curves of the World Health Organization with the use of the software WHO-Anthro Plus 2007 [31]. The values obtained will be classified according to the cutoff points recommended by the WHO for children over 5 years of age, being underweight: below –2Z; eutrophy: between –2 and + 1Z; overweight: between + 1 and + 2Z; obesity between + 2Z and + 3Z and severe obesity above 3Z of the reference median. This classification will be performed at baseline to confirm the inclusion criteria.

#### Secondary outcomes

Blood pressure will be measured with an electronic and digital device Omron® Hem 742, previously validated for use in children [32]. Before taking the measurement, it will be certified that the child or adolescent does not have a full bladder, has not ingested medications and/or coffee, has not eaten up to 30 min before the procedure and/or practiced physical exercises up to 1 h before. The measurement will be performed on the right arm, at the same level as the heart, supported on a table with a flat surface, with the palm of the hand facing up and the elbow slightly flexed. The individual should be seated, with feet in contact with the floor or flat surface platform, and remain in this position for 5 min at rest before the measurement [33]. Two BP measurements will be performed, with a minimum interval of 2 min, and the mean of the 2 measurements will be calculated. If the difference between the 2 measurements is greater than or equal to 5 mmHg, a new measurement will be taken.

The other secondary outcomes of the study investigated will be variations in the BMI of the responsible individual, waist circumference (WC), waist height ratio (WHtR), and urinary sodium/potassium.

The adult BMI is obtained through weight (kg) divided by height (m) squared. The values obtained will be classified according to the cutoff points recommended by the WHO for adults over 18 years of age, being underweight: below 18.5; eutrophy: between 18.6 and 24.9; overweight: between 25.0 and 29.9; grade I obesity: between 30.0 and 34.9; grade II obesity: between 35.0 and 39.9 and grade III obesity: above 40.0.

For the measurement of WC, a flexible and inelastic tape measure will be used, with an amplitude of 150 cm and a variation of 0.1 mm. WC will be measured with tape placed horizontally at the midpoint between the lower edge of the last rib and the iliac crest. The measurements will be performed with the tape firm on the skin, however, without compression of the tissues, being the evaluated standing with relaxed abdomen and with the arms extended to the side of the body. For the QC, the tape measure will be placed horizontally around the hip in the most protruding part of the buttocks [34]. The WHtR will be obtained by the ratio between WC and height, both in centimeters.

To measure urinary sodium and potassium, the spot urine method will be used [35]. A clean and sterile bottle will be provided. The participant will be instructed to open the bottle only at the time of collection to avoid contamination. The pot with the urine collected at any time of the day will be packed in Styrofoam and taken to the laboratory.

### Measures of adherence to intervention

Food consumption will be evaluated by the Food Frequency Questionnaire administered at baseline and at the end of the study. Based on the responses, individual goals will be created, and the intervention group will receive a calendar that aims to monitor the fulfillment of these established goals.

The physical activity of the guardians will be assessed using the IPAQ-SF [36] and children using the PAQ-C [37]. In addition, moderate-to-vigorous physical activity and sedentary time for children will be assessed using triaxial accelerometers (ActiGraph GT3x-BT, Pensacola, FL, USA) [38] positioned on the nondominant wrist for 7 consecutive days at baseline and after the intervention period. The adherence to the exercise protocol will be evaluated through a questionnaire at the end of the intervention, where participants should report how many classes per week were performed.

### Ethical aspects

This study was reviewed and approved by the Research Ethics Committee of the Instituto de Medicina Social Hésio Cordeiro, State University of Rio de Janeiro (CAAE 59008422.8.0000.5260). This is an IRB (institutional review board). Informed consent will be obtained from all participants and their legal guardians by signing the consent form of guardians and children's consent, pursuant to Resolution nº 466/2012.

The study protocol is registered in the virtual platform of free access for registration of experimental and nonexperimental studies conducted in humans and conducted in Brazilian territory, the Brazilian Registry of Clinical Trials (RBR-10 mm62vs). The results of the study will be presented according to the guidelines of the consolidated report for clinical studies – CONSORT (Consolidated Standards of Reporting Trials) [39].

## Discussion

The project will test the effectiveness of the use of the recommendations of the PHD, physical activity and a salt-reduced sodium. The results of the present study will allow the refinement of interventions aimed at the treatment of childhood obesity and may help develop guidelines for the treatment of obesity in Brazilian children.

In recent decades, family-based interventions have shown promise in weight control in children, but this type of intervention is not yet incorporated into primary care for the treatment of childhood obesity. The proposal aims to simplify the counseling regarding diet and practice of physical activity, since often in primary care, there is no figure of the nutritionist and physical education professional to calculate the eating and physical activity plan.

Another advantage of the proposal is the guidance based on the PHD, a promising strategy to reduce weight gain by changing eating behavior, which promotes sustainable and conscious use of the planet's resources. The EAT-Lancet recommendation applied to the Brazilian diet suggested less harm to the planet, higher nutritional quality of the diet and possible reduction of BMI.

Intake of salt is the major contributor of diet to mortality by noncommunicable diseases (NCDs) in Brazil, and NCDs are one of the most important public health problems in Brazil [40]. A previous intervention in a primary care setting in Northeast Brazil using a combination of regular salt added with herbs and nutritional guidance to reduce products with a high density of sodium was effective in reducing the blood pressure of hypertensive adults [27]. In this study, a positive finding under the hypothesis of the effectiveness of blood pressure reduction by the modified salt may bring great advantages for the prevention of hypertension, as it is a simple and low-cost strategy that may have a great impact on health. Epidemiological studies suggest that high sodium intake may also be associated with an increased prevalence of obesity [41, 42], so reducing sodium intake may aid in weight loss.

One of the motivations for including the factorial design was to increase the adherence of participants to treatment. The salt was well accepted according to a sensory test in a small sample. Additionally, in many trials for the treatment of obesity, after 3 months of intervention, there is weight regain and loss to follow-up. The short follow-up period reduces drop out. Moreover, there is a possibility that reducing the focus on weight change encourages participants to continue treatment.

Therefore, we expect to demonstrate that the multicomponent family-based intervention is operationally feasible and confers weight loss and reduction of blood pressure levels in children and their guardians. The results of the present study could allow the refinement of intervention studies aimed at the treatment of childhood obesity.

## Data Availability

The datasets generated and/or analyzed during the current study are available from the corresponding author on reasonable request.

## Abbreviations

BMI: Body Mass Index
BP: Blood Pressure
CONSORT: Consolidated Standards of Reporting Trials
DASH: Dietary Approaches to Stop Hypertension
ELSA: Longitudinal Study of Adult Health
FFQ: Food Frequency Questionnaire
HDL-c: High Density Lipoprotein Cholesterol
IPAQ-SF: International Physical Activity Questionnaire - Short Form
IRB: Institutional Review Board
KCL: Potassium Chloride
LDL: Low-density Lipoprotein
NCDs: Noncommunicable diseases
PAQ-C: Physical Activity Questionnaire for Children
PHD: Planetary healthy diet
PHDI: Brazilian Planetary Health Diet Index
RBR: Brazilian Registry of Clinical Trials
SAS: Statistical Analysis System
WC: Waist Circumference
WHO: World Health Organization
WHtR: Waist Height Ratio
